# Supplementary material for: Nucleocytoplasmic transport of active HER2 causes fractional escape from the DCIS-like state
Source: Nat Commun. 2023 Apr 13;14:2110. doi: 10.1038/s41467-023-37914-x (PMC10102026; doi:10.1038/s41467-023-37914-x)
Supplement: Supplementary file 1 — Supplementary Information [file 41467_2023_37914_MOESM1_ESM.pdf]

SUPPLEMENTARY INFORMATION

Nucleocytoplasmic transport of active HER2 causes fractional escape from the DCIS-like state

Lixin Wang[1]*, B. Bishal Paudel[1]*, R. Anthony McKnight[1,2], Kevin A. Janes[1,3]✉

[1]Department of Biomedical Engineering, University of Virginia, Charlottesville, Virginia, USA.
[2]Olympus Veran Technologies, St. Louis, Missouri, USA.
[3]Department of Biochemistry & Molecular Genetics, University of Virginia, Charlottesville, Virginia, USA.
*These authors contributed equally: L. Wang, B.B. Paudel.
✉Email: kjanes@virginia.edu

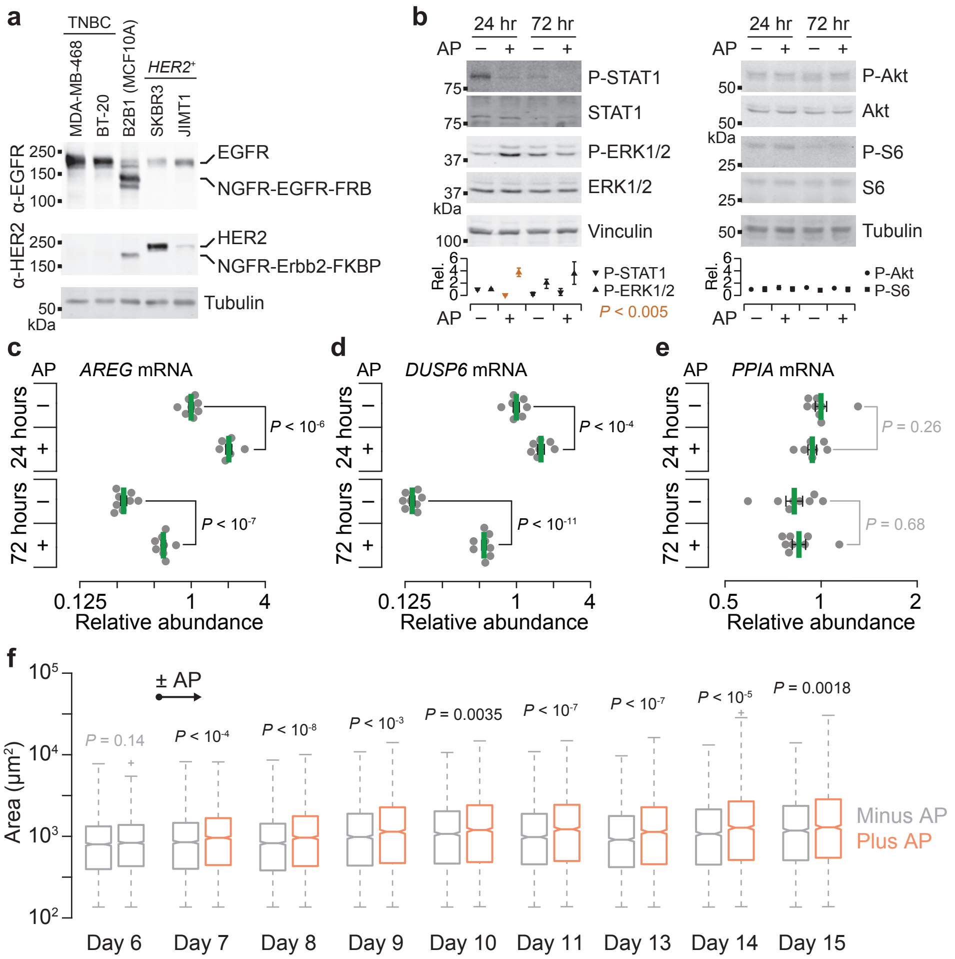

Supplementary Fig. 1 | A B2B1 subclone of MCF10A-5E cells with moderate ectopic expression of chemically activatable EGFR and Erbb2 chimeras.

a, Abundance of NGFR-EGFR-FRB and NGFR-Erbb2-FKBP chimeras in B2B1 cells is comparable to endogenous expression in triple-negative breast cancer (TNBC) and *HER2*-amplified (*HER2*$^+$) breast cancer lines respectively. Protein extracts were immunoblotted for EGFR and HER2 intracellular domains with tubulin used as a loading control.

b, ErbB heterodimerization alters phosphorylation of STAT1 (Tyr701) and ERK1/2 (Thr202/Tyr204) but not phosphorylation of Akt (Ser473) or S6 (Ser240/244). Protein extracts from 3D-cultured B2B1 cells treated ±0.5 μM AP21967 (AP) for the indicated times after Day 6 were immunoblotted for the indicated proteins with total STAT1, total ERK1/2, total Akt, total S6, vinculin, and tubulin used as loading controls. Bottom graphs show the mean ± range of replicated densitometry from n = 3 biological replicates; at each time point, differences between ±AP conditions were assessed by two-sided t test.

c–e, ErbB heterodimerization induces canonical ErbB target genes with no detectable effect on housekeeping genes. Quantitative PCR for *AREG* (**c**), *DUSP6* (**d**), and *PPIA* (**e**) in B2B1 cells cultured in 3D for six days ±0.5 μM AP21967 (AP) for 24 hours or 72 hours where indicated. Data are shown as the geometric mean (normalized to the 24-hour, minus-AP condition) ± log-transformed s.e. from n = 7 (plus-AP, 24 hours) or 8 biological replicates (all other groups). Differences in geometric means were assessed by two-sided t test after log transformation.

f, ErbB heterodimerization causes a global and sustained increase in outgrowth size within 24 hours of AP addition. B2B1 cells were cultured in 3D for six days followed by stimulation with or without 0.5 μM AP for the indicated times after Day 6. Cross-sectional areas were quantified for n = (left to right) 2299, 2317, 2154, 2176, 2111, 2089, 2011, 2011, 1988, 1988, 1934, 1928, 1921, 1899, 1909, 1821, 1851, or 1774 outgrowths from four biological replicates. Boxplots show the median area, estimated 95% confidence interval of the median (notches), interquartile range (box), 1.5x the interquartile range from the box edge (whiskers), and outliers (+). Differences between groups for each time point were assessed by two-sided rank sum test with Šidák correction for multiple-hypothesis testing.

Source data are provided as a Source Data file.

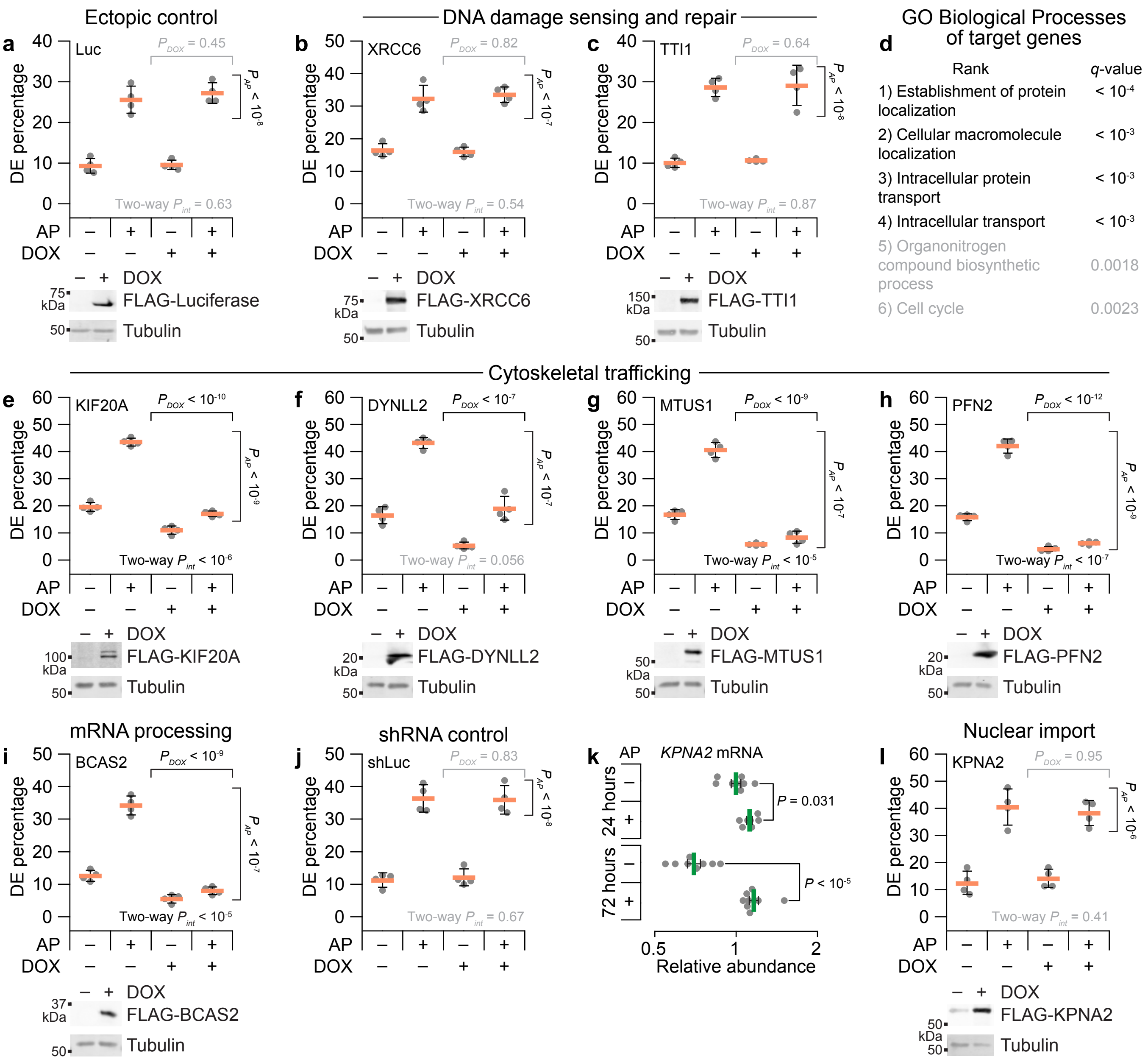

Supplementary Fig. 2 | Exclusion of alternative hypotheses about other candidates.

a, DCIS escapee (DE) percentage for B2B1 cells stably expressing inducible FLAG-tagged luciferase.

b, **c**, Candidates related to DNA damage sensing–repair do not affect DE penetrance. B2B1 cells expressing inducible FLAG-tagged XRCC6 (**c**) or TTI1 (**d**) were used where indicated.

d, Enrichment analysis of gene ontology (GO) biological processes for the 97 candidate genes (= 15 high-priority targets + 86 candidates – [4 uncharacterized loci and probeset redundancies]) identified by stochastic frequency matching. False discovery rate-corrected q-values are shown for each biological process.

e–**h**, Candidates related to cytoskeletal trafficking inhibit ErbB-induced DE penetrance. B2B1 cells expressing inducible FLAG-tagged KIF20A (**e**), DYNLL2 (**f**), MTUS1 (**g**), or PFN2 (**h**) were used where indicated.

i, A candidate amplified in breast cancer[1] and involved in mRNA processing inhibits ErbB-induced DE penetrance. B2B1 cells expressing inducible FLAG-tagged BCAS2 were used.

j, DE percentage for B2B1 cells stably expressing inducible shRNA targeting luciferase (shLuc) as an shRNA control.

k, Quantitative PCR for *KPNA2* in B2B1 cells cultured in 3D for six days followed by addition of 0.5 μM AP21967 (AP) for the indicated times. Data are shown as the geometric mean (normalized to the 24-hour, minus-AP condition) ± log-transformed s.e. from n = 7 (plus-AP, 24 hours) or 8 biological replicates (all other groups). Differences in geometric means were assessed by two-sided t test after log transformation.

l, A regulator of nuclear import does not affect ErbB-induced DE penetrance. B2B1 cells expressing inducible FLAG-tagged KPNA2 were used.

For **a–c**, **e–j**, and **l**, B2B1 cells stably expressing doxycycline (DOX)-inducible ectopic constructs were 3D cultured for 9–13 days with 0.5 μM AP added at Day 6 and/or 1 μg/ml DOX added at Day 5 where indicated. Data are shown as the arcsine transformed mean ± s.e. from n = 4 biological replicates where >100 outgrowths were scored per replicate. Differences by factor (DOX or AP) and two-factor interaction (int) were assessed by two-way ANOVA after arcsine transformation. Ectopic expression was confirmed by immunoblotting for FLAG with tubulin used as a loading control.

Source data are provided as a Source Data file.

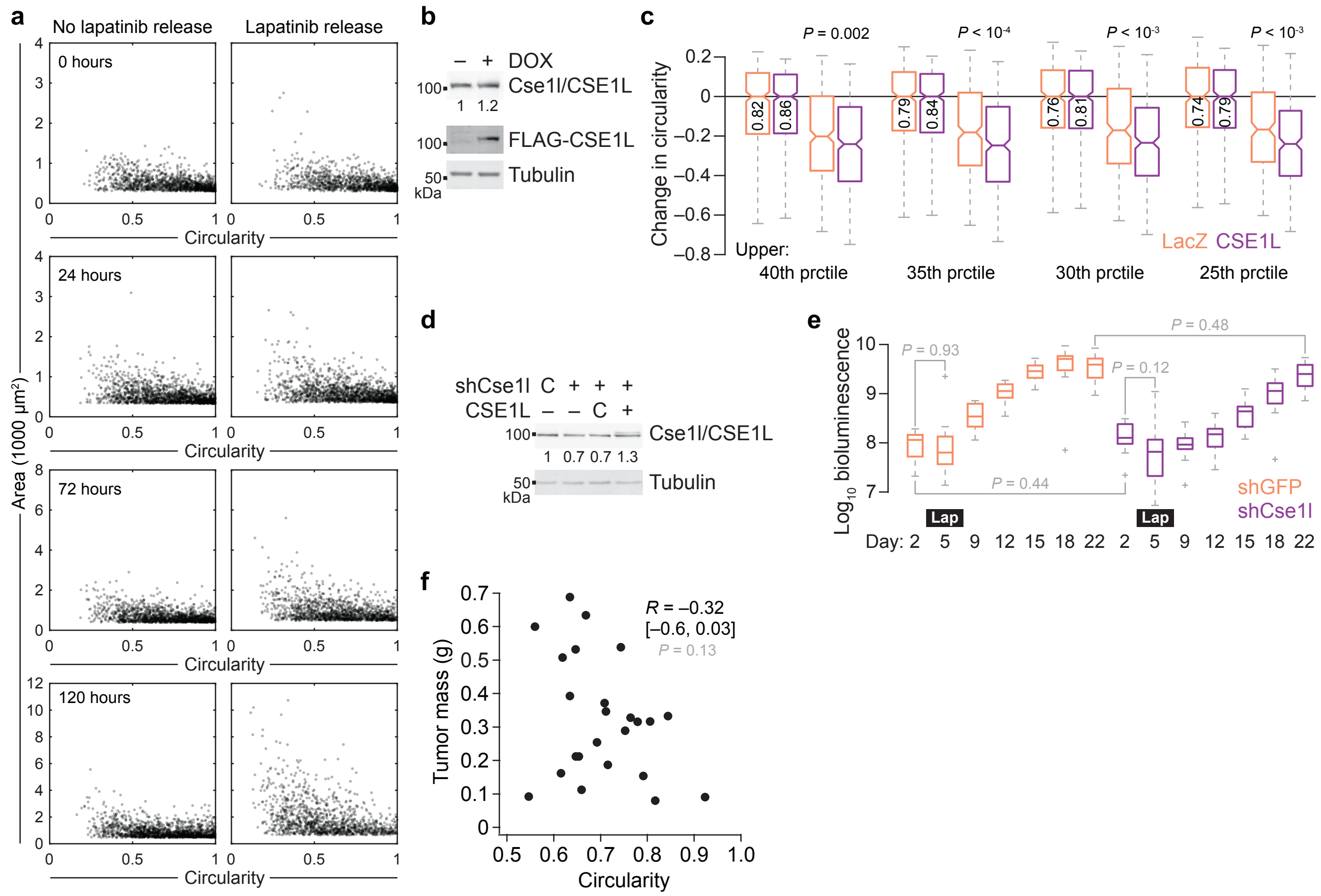

Supplementary Fig. 3 | 3D and in vivo characterization of TM15c6 cells with or without pharmacologic and genetic perturbations.

a, Changes in cross-sectional area upon lapatinib release do not noticeably skew changes in circularity. TM15c6 cells 3D cultured for five days in 2 μM lapatinib followed by release for the indicated time. Cross-sectional area and circularity were analyzed for all outgrowths [*n* = (horizontally then vertically) 2026, 2150, 2639, 2637, 2163, 2455 1816, or 2341 outgrowths from four biological replicates].

b, Inducible ectopic expression of CSE1L confirmed by immunoblotting for total Cse1l/CSE1L and FLAG with tubulin used as a loading control. Inducible overexpression of total Cse1l/CSE1L was quantified relative to the minus-DOX control.

c, CSE1L-induced circularity phenotype is not sensitive to the choice of cross-sectional area thresholding. Circularities were analyzed for outgrowths above the indicated threshold by size [*n* = (left to right) 1061, 1364, 958, or 1180 outgrowths from four biological replicates].

d, Inducible knockdown of Cse1l and addback of CSE1L. TM15c6 cells stably expressing inducible shCse1l or shGFP control (C) with or without inducible CSE1L or LacZ control (C) were treated with 1 μg/ml doxycycline for two days and immunoblotted for Cse1l/CSE1L with tubulin used as a loading control.

e, Dynamics of tumor bioluminescence in TM15c6 cells expressing shCse1l or shGFP control after inoculation at Day 0 and administration of 30 mg/kg lapatinib (Lap) i.p. daily from Days 3–9 (*n* = 12 animals).

For **c** and **e**, boxplots show the median bioluminescence, interquartile range (box), estimated 95% confidence interval of the median (**c**, notches), 1.5x the interquartile range from the box edge (whiskers), and outliers (+). Differences between indicated groups were assessed by two-sided rank sum test with Šidák correction for multiple-hypothesis testing.

f, Tumor size is not significantly correlated with tumor circularity. Mass and circularity of excised tumors (*n* = 24 samples) were assessed by Pearson correlation (*R*) with 95% confidence interval and hypothesis testing for nonzero correlation estimated after Fisher Z transformation.

Source data are provided as a Source Data file.

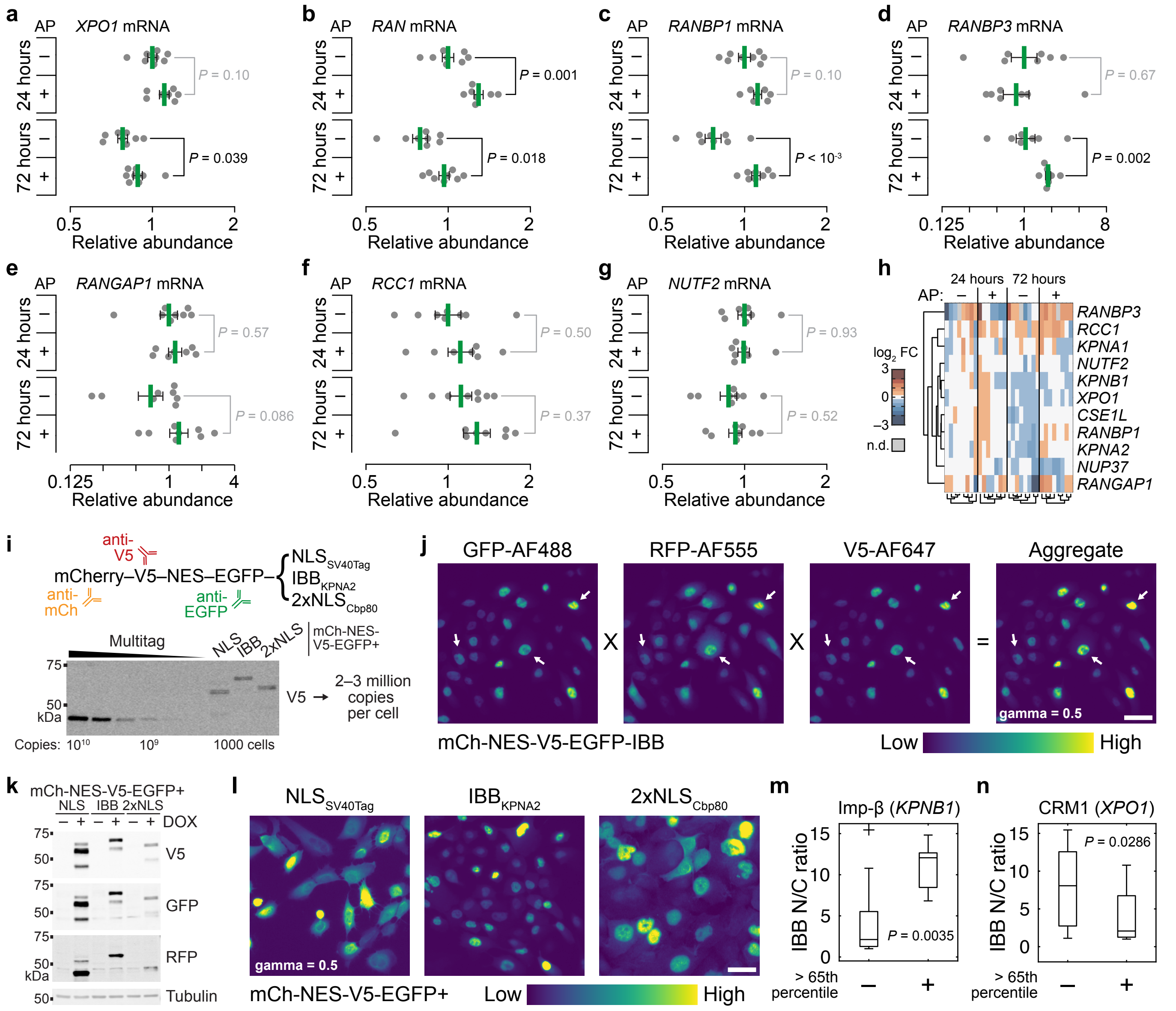

Supplementary Fig. 4 | Motivation, testing, and use of a nucleocytoplasmic transport model.
a–g, Quantitative PCR for *XPO1* (**a**), *RAN* (**b**), *RANBP1* (**c**), *RANBP3* (**d**), *RANGAP1* (**e**), *RCC1* (**f**), and *NUTF2* (**g**) in B2B1 cells cultured in 3D for six days followed by 0.5 µM AP21967 (AP) for 24 hours or 72 hours. Data are shown as the geometric mean (normalized to the 24-hour, minus-AP condition) ± log-transformed s.e. from *n* = 7 (plus-AP, 24 hours) or 8 biological replicates (all other groups). Differences in geometric means were assessed by two-sided *t* test after log transformation.
h, Replicate-by-replicate clustergram of the quantitative PCR data from Fig. 2g–j, Supplementary Fig. 2k, and Supplementary Fig. 4a–g. Data were normalized to the geometric mean of the 24-hour, minus-AP condition for each gene and row clustered (Euclidean distance, Ward's linkage). Replicate groups were column clustered separately
i, Tandem cargo reporters. mCherry (mCh), V5, and EGFP track steady-state accumulation of different NLSs paired with an NES. Absolute copy-number quantification was performed with recombinant V5-containing Multitag[2].
j, Image math for Alexa Fluor (AF)-based immunolocalization of GFP, RFP/mCh, and V5 to estimate full-length tandem cargo reporter.
k, Inducible expression of tandem cargo reporters. B2B1 cells expressing inducible mCh-NES-V5-EGFP tagged with NLS$_{SV40Tag}$, IBB$_{KPNA2}$, or 2xNLS$_{Cbp80}$ induced with 1 µg/ml doxycycline (DOX) for 6 hours were immunoblotted for the indicated epitopes with tubulin used as a loading control.
l, Representative image math immunolocalization of the indicated tandem cargo reporters after DOX induction for 6 hours. Quantification is summarized in Fig. 3b–d.

m, **n**, Proportionately high expression of Imp-β and CRM1 alters the steady-state accumulation of Imp-β-binding cargo. Predicted IBB nuclear/cytoplasmic (N/C) ratio for n = 20 AP-treated B2B1 outgrowths split at the 65th percentile according to relative transcript abundance of *KPNB1* (**e**), or *XPO1* (**f**). Boxplots show the median N/C ratio (horizontal line), interquartile range (box), 1.5x the interquartile range from the box edge (whiskers), and outliers (+). Differences between groups were assessed by a one-sided rank sum test.
For **j** and **l**, aggregate images are displayed with image gamma = 0.5, and the scale bar is 20 μm.
Source data are provided as a Source Data file.

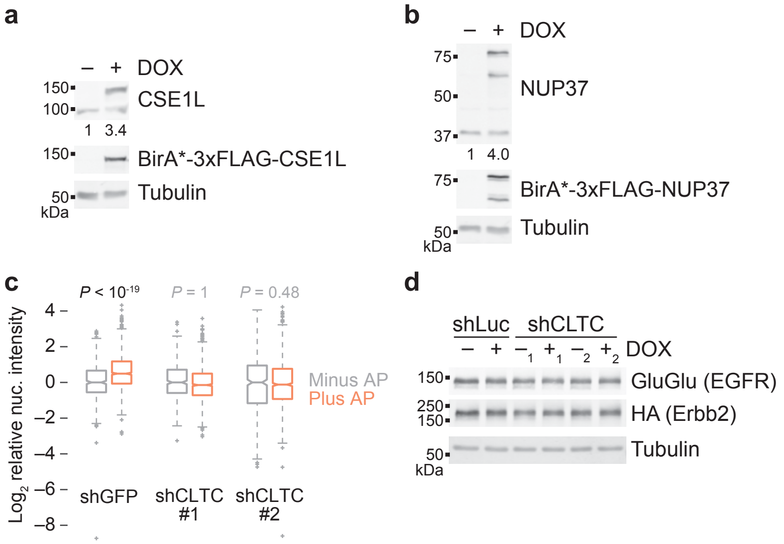

Supplementary Fig. 5 | Extended data related to CSE1L–NUP37 proximity labeling and CLTC knockdown.
a, b, Induction of ectopic BirA*-CSE1L (**a**) or BirA*-NUP37 (**b**) relative to endogenous protein. Inducible expression was compared relative to the no DOX control with tubulin used as a loading control.
c, CLTC knockdown inhibits the internalization of EGFR–Erbb2 heterocomplexes. B2B1 cells expressing the indicated shRNAs were plated on coverslips and induced with 1 μg/ml DOX for 48 hours before treatment with 0.5 μM AP for 15 minutes and immunostaining for GluGlu and HA tags. Boxplots show the normalized nuclear intensity (relative to minus AP), interquartile range (box), estimated 95% confidence interval of the median (notches), 1.5x the interquartile range from the box edge (whiskers), and outliers (+) from n = (left to right) 884, 1199, 433, 1146, 434, or 1017 cells of two or four biological replicates. Differences between AP groups were assessed by one-sided rank sum test.
d, CLTC knockdown does not alter long-term abundance of EGFR or Erbb2 chimeras. B2B1 cells expressing the indicated shRNAs were immunoblotted for GluGlu and HA tags with tubulin used as a loading control.
Source data are provided as a Source Data file.

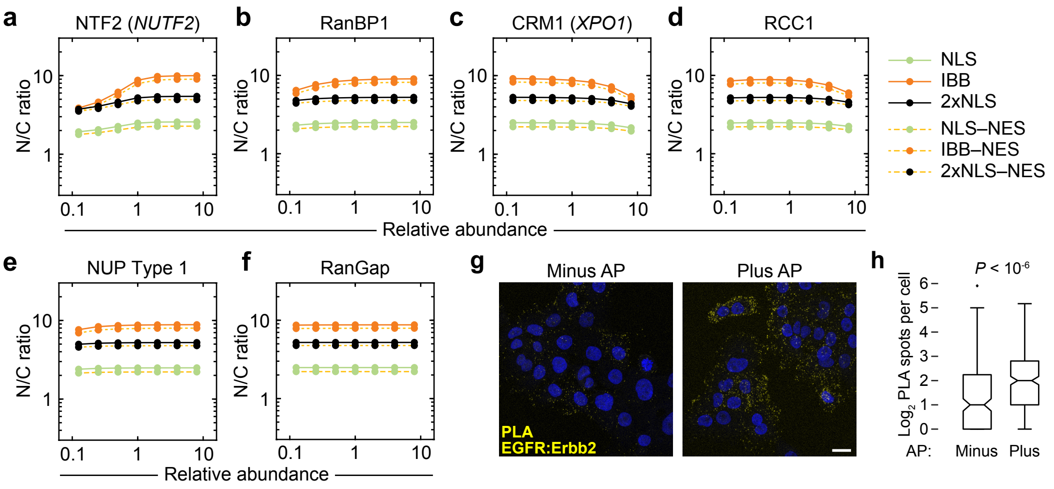

Supplementary Fig. 6 | Extended sensitivity analysis of the model and validation of the proximity ligation assay.

a–f, Predicted sensitivity of the systems model to starting protein concentrations of NTF2 (**a**), RanBP1 (**b**), CRM1 (**c**), RCC1 (**d**), total NUP (**e**), and RanGap (**f**). Protein abundances are scaled relative to the concentrations used in the base model (Supplementary Data 4). Steady-state nuclear-to-cytoplasmic (N/C) ratios are shown for 1 μM of the representative cargo described in Fig. 4a.

g, The proximity ligation assay detects regulated protein-protein interactions. B2B1 cells were treated with or without 0.5 μM AP for 15 minutes and costained for interaction between the EGFR and Erbb2 chimeras. Scale bar is 20 μm.

h, Quantification of PLA spots in B2B1 cells treated with or without 0.5 μM AP and stained as in (**g**). Boxplots show the median PLA spots per cell (horizontal line), interquartile range (box), estimated 95% confidence interval of the median (notches), 1.5x the interquartile range from the box edge (whiskers), and outliers (+) from n = (left to right) 195 or 272 cells from eight confocal image stacks on one coverslip per condition. Differences between groups were assessed by two-sided KS test with Bonferroni correction.

Source data are provided as a Source Data file.

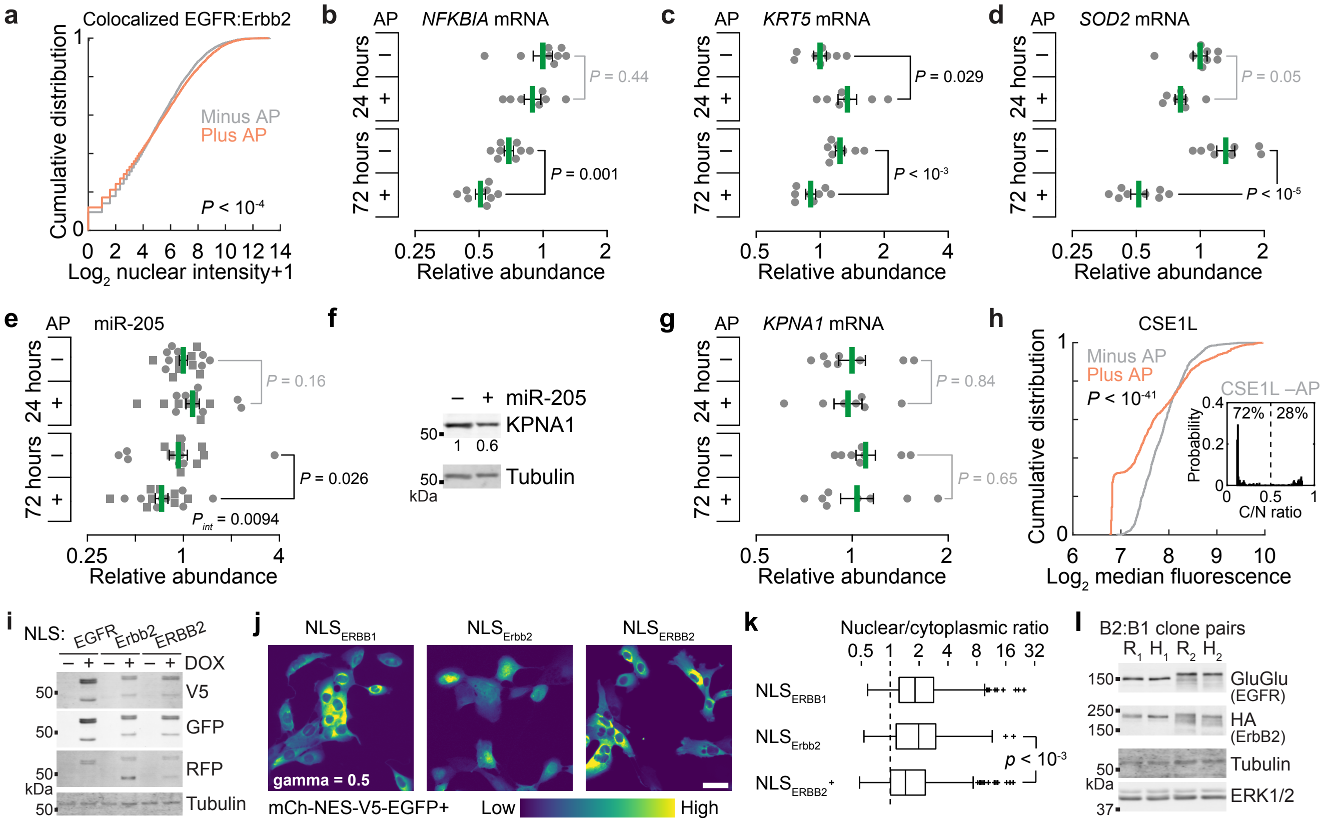

Supplementary Fig. 7 | Genomic loci bound by chimeric EGFR, controls for miR-205, and comparisons of ErbBs from primates and rodents.

a, Colocalized nuclear EGFR:Erbb2 staining in B2B1 cells cultured in 3D for six days ±0.5 μM AP for 24 hours. Differences were assessed by two-sided KS test.

b–e, Quantitative PCR for *NFKBIA* (**b**), *KRT5* (**c**), *SOD2* (**d**), and miR-205 (**e**) in B2B1 cells cultured in 3D for six days ±0.5 μM AP21967 (AP) for 24 hours or 72 hours.

f, miR-205 suppresses KPNA1. 293T cells were transfected with miR-205 precursor or negative control for 48 hours and immunoblotted for KPNA1 with tubulin used as a loading control.

g, Quantitative PCR for *KPNA1* in B2B1 cells cultured in 3D for six days ±0.5 μM AP21967 (AP) for 24 hours or 72 hours where indicated.

h, Median CSE1L immunoreactivity after 3D culture for six days ±0.5 μM AP for 24 hours. Data show the cumulative distribution from *n* = 894 (–AP) or 870 (+AP) cells from four biological replicates. Differences were assessed by two-sided KS test. (Inset) Model predictions (Fig. 7k) when CSE1L is reduced to 40% (reflecting 3D culture; Fig. 2g) and randomly perturbed (**h**, gray). Simulations were performed as in Fig. 7k with K_D = 3 nM, and steady-state cytoplasmic/nuclear (C/N) ratios above or below 50% are shown from *n* = 500 initializations.

i, Inducible ErbB tandem cargo reporters validated as in Supplementary Fig. 4k.

j,k, Representative immunolocalization (**j**) and quantification (**k**) after DOX induction for 6 hours. Boxplots show the median N/C ratio (horizontal line), interquartile range (box), 1.5x the interquartile range from the box edge (whiskers), and outliers (+) from *n* = 283 (NLS_{ERBB1}), 285 (NLS_{Erbb2}), and 333 (NLS_{ERBB2}) cells collected from four biological replicates. Differences were assessed by two-sided rank sum test. For **j**, the scale bar is 20 μm.

l, Characterization of ErbB clones expressing human ERBB2 or rat Erbb2. Protein extracts were immunoblotted for GluGlu and HA epitope tags with tubulin and ERK1/2 used as loading controls.

For **b–e** and **g**, data are shown as the geometric mean (normalized to the 24-hour, minus-AP condition) ± log-transformed s.e. from *n* = 7 (plus-AP, 24 hours) or 8 biological replicates (all other groups). Differences in geometric means were assessed by two-sided *t* test after log transformation.

Source data are provided as a Source Data file.

Supplementary Note 1. Development of the nucleocytoplasmic transport model.

Model Reconstruction

The nucleocytoplasmic transport model was based upon two earlier models of Riddick and Macara[3, 4]. The first model[3] encodes importin-α/β shuttling dynamics, the import of cargo harboring different types of nuclear localization sequence (NLS), and the transport of Ran. Code for the first model was obtained from the original publication as a Jarnac script[5], which we converted to MATLAB language with a Jarnac-to-MATLAB translator from the Systems Biology Workbench[6]. This model recreated most of the simulations reported in the publication[3] but required multiple edits to reflect accurately the experimental parameters drawn from the literature (see Model Corrigenda below).

The second model[4] builds upon the first by adding the machinery for nuclear export along with a dedicated subcompartment for nuclear pores. Regrettably, code for the second model could not be recovered from the BioModels database[7] where it was purportedly deposited[4]. As an alternative, we recoded the second model to the extent possible given the descriptions and parameters reported in Ref. [4]. The recoding required some assumptions that are elaborated upon here (see Nuclear Pore Complex, Nuclear Export Module, and Model Addenda below). The final model reflecting all changes and additions has been deposited in the BioModels database (MODEL2210060001) and is available on GitHub (JanesLab/NucCytoShuttle).

Model Corrigenda

Although the MATLAB translation of the Ref. [3] model was accurate, we uncovered multiple inconsistencies in reported parameter values after checking the original sources. Some were errors in transcription from the literature. For example, one cited study[8] quantifying the interaction between RanGTP and importin-β reports a half-life ($t_{1/2}$) of four hours for the RanGTP:importin-β complex. By definition, $t_{1/2}$ is related to the dissociation rate constant (k_{off}) as follows:

$$k_{off} = \frac{\ln(2)}{t_{1/2}} \tag{1}$$

yielding k_{off} = 4.8 x 10^{-5} s^{-1} for the RanGTP:importin-β complex. However, in Ref. [3] and in the original Jarnac code, this dissociation rate constant is listed as 4.8 x 10^{-6} s^{-1}, giving rise to a tenfold higher affinity in the model than that reported in Ref. [8]. In the same table of Ref. [3], association rate constants (k_{on}) are listed in units of $\mu M^{-1}s^{-1}$ but the values in the Jarnac code support units of $M^{-1}s^{-1}$, which are also more biophysically realistic. Likewise, bimolecular-to-biomolecular exchange reactions—such as importin-α–importin-β–NLS + RanGTP ↔ importin-α–NLS + importin-β–RanGTP—should have reverse rates in units of $M^{-1}s^{-1}$, not s^{-1} as listed[3]. We corrected these parameters and others with evidence of transcription errors (Supplementary Data 4).

Elsewhere, we discovered inconsistencies in the reporting of model parameters. Ref. [3] lists parameters for the RanGAP-catalyzed hydrolysis of a RanGTP–CAS–importin-α–RanBP1 species, but this species does not exist in the Jarnac code. Instead, RanBP1 binding to RanGTP–CAS–importin-α converts to RanBP1–RanGTP + CAS + importin-α, which we retain in the final model. Estimates of other transport receptors and native cargo for importin-α–importin-β and importin-β are listed as whole-cell concentrations in Ref. [3] but used as cytoplasmic concentrations in the Jarnac code. We use the Ref. [3] numbers as whole-cell concentrations to stay consistent with the other proteins in the same table. The accumulating corrections and changes prompted us to abandon attempts at retaining all the modeling results of the earlier work.

Most problematic was the overall handling of interactions (importin-α–importin-β, importin-α–importin-β–NLS, and importin-β–cargo from Ref. [3]) that were modeled using two-state kinetics estimated from surface-plasmon resonance data of Catimel et al.[9]. A two-state model casts the bimolecular interaction of *A* and *B* as:

$$A + B \underset{k_{off,1}}{\overset{k_{on,1}}{\rightleftarrows}} AB^* \underset{k_{off,2}}{\overset{k_{on,2}}{\rightleftarrows}} A{:}B \tag{2}$$

where *AB** is a precursor complex, and *A:B* is the mature complex. Unlike a one-state association rate constant, $k_{on,2}$ is defined in units of per time. Refs. [3, 9] both list $k_{on,2}$ in units of per concentration per time. The difference is crucial because of the way that an effective dissociation constant ($K_{D,eff}$) is defined for a two-state model:

$$K_{D,eff} = \frac{k_{off,1}}{k_{on,1}\left(1+\frac{k_{on,2}}{k_{off,2}}\right)} \quad (3)$$

Ref. [9] lists the four rate parameters of the two-state model along with an estimated $K_{D,eff}$, which according to Equation 3 is incorrect by roughly one million-fold (~fM affinity instead of ~nM). The parameter table in Ref. [9] lists $k_{on,2}$ in units of 10^3 $M^{-1}s^{-1}$—based on one text reference to the $k_{on,2}$ for the importin-β:cargo complex, we believe all of the $k_{on,2}$ values are instead in units of 10^{-3} s^{-1}. Changing the units in this way reconciles the two-state kinetic parameters of Ref. [9] with the accompanying estimate of $K_{D,eff}$ and implies that all $k_{on,2}$ parameters in Ref. [3] are incorrect by six orders of magnitude (Table SN1). The final model uses the revised set of parameters that are self-consistent with the original data source[9] (Supplementary Data 4).

Nuclear Pore Complex

To model nuclear pore complexes (NPCs), we began by creating a perinuclear subcompartment comprised of volume removed from the nuclear and cytoplasmic subcompartments. The latest cryoelectron microscopy data on the human NPC suggest that it is 70 nm tall[10, 11]. Using this dimension (h_{NPC}) and the volume of the nucleus specified in the model (V_{nuc}), we approximated the nucleus as a sphere and removed radial volume from the nucleus and cytoplasm to create the perinuclear volume (V_{pn}):

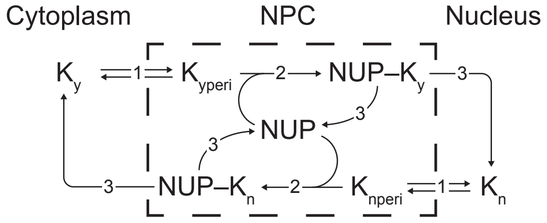

Fig. SN1. Modeling architecture for the transport of karyopherins (K) and karyopherin-containing complexes across the NPC subcompartment through a nuclear pore (NUP).

$$V_{pn} = \frac{4\pi}{3}\left[\left(\sqrt[3]{\frac{3}{4\pi}V_{nuc}} + \frac{h_{NPC}}{2}\right)^3 - \left(\sqrt[3]{\frac{3}{4\pi}V_{nuc}} - \frac{h_{NPC}}{2}\right)^3\right] \quad (4)$$

We then updated the volumes for the nucleus ($V_{nuc\text{-}pc}$) and cytoplasm ($V_{cyto\text{-}pc}$) accordingly:

$$V_{nuc-pc} = \frac{4\pi}{3}\left(\sqrt[3]{\frac{3}{4\pi}V_{nuc}} - \frac{h_{NPC}}{2}\right)^3 \quad (5)$$

$$V_{cyto-pc} = V_{cyto} + V_{nuc} - \frac{4\pi}{3}\left(\sqrt[3]{\frac{3}{4\pi}V_{nuc}} + \frac{h_{NPC}}{2}\right)^3 \quad (6)$$

Last, V_{pn} was populated with cellular estimates of NPC species (see Model Addenda and Model Generalization below), which were confined to the perinuclear subcompartment.

Karyopherin-mediated transport through V_{pn} was modeled in the following directional sequence: 1) Cytoplasmic (or nuclear) karyopherin exchanges with the perinuclear subcompartment at the rate used to describe karyopherin-independent nucleocytoplasmic transport in the original model[3]. 2) Perinuclear karyopherin binds to an unoccupied nuclear pore (NUP) according to the kinetic parameters reported in Ref. [4]. 3) The NUP:karyopherin complex unbinds, releasing karyopherin into the nuclear (or cytoplasmic) subcompartment and retaining the dissociated NPC in the perinuclear subcompartment (Fig. SN1). The final model leaves open the possibility of two classes of nuclear pores[12], one that transports all cellular cargo and one that additionally transports a specific cellular cargo. The overall formalism achieves bidirectional transport across the NPC and Identifies when import-export kinetics are limited by the number of available NPCs per cell. In the final model, NPC limitations occur with fewer than ~10 cargo-specific nuclear pores per cell (Fig. SN2). These modeling results are consistent with the enormous cargo-carrying capacity of individual NPCs[13].

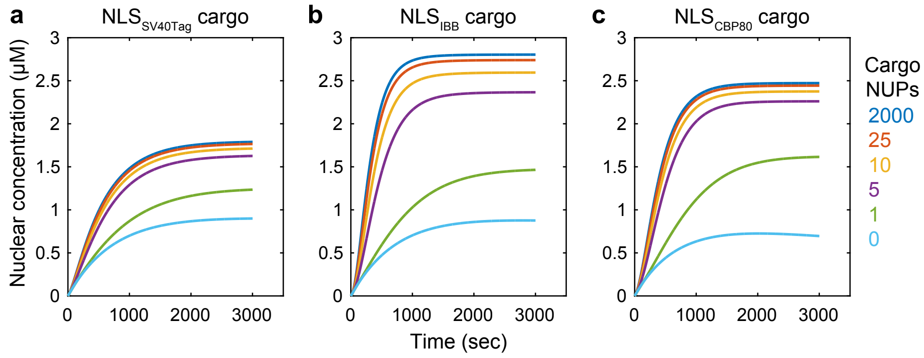

Fig. SN2. Evidence for limitations in cargo transport when nuclear pores (NUPs) are extremely limiting. Details on the different NLS cargos are explained later in the text. Simulations were performed by spiking in cargo at a whole-cell concentration of 1 µM.

Nuclear Export Module

The nuclear export module of Ref. [4] adds three proteins: a

prototypical NES-containing cargo, the exportin CRM1, and the cofactor RanBP3 that enhances CRM1-mediated export[14, 15]. NES cargo, CRM1, and RanBP3 assemble with RanGTP in the nucleus, and the four-protein holocomplex is exported through the NPC according to the transport mechanisms described above. In the cytoplasm, CRM1–RanBP3–NES–RanGTP binds to RanBP1, which enables RanGAP to promote GTP hydrolysis and dissociation of the holocomplex into its individual constituents. RanBP3 contains a conventional NLS, which is reimported into the nucleus via importin-α/β, and CRM1 passively exchanges between the cytoplasm and nucleus by karyopherin-independent diffusion.

Although Ref. [4] is unclear about the kinetic parameters for recycling RanBP3 and CRM1 to the nucleus and for assembling–disassembling CRM1–RanBP3–NES–RanGTP, we managed to fill voids with existing parameters by making reasonable assumptions. For example, formation of the CRM1 and RanBP3 intermediate complexes with RanGTP were assumed to occur with the same kinetics as the association kinetics listed in Ref. [4] for CRM1–RanBP3 + RanGTP. Likewise, we assumed that the RanBP1-induced disassembly of CRM1–RanBP3–NES–RanGTP was the same as that for RanGTP–CAS–importin-α in Ref. [3] (see Model Corrigenda above). The NLS on RanBP3 was assumed to bind with the same kinetics as $NLS_{SV40Tag}$, and the permeability of CRM1 was assumed to be the same as CAS (another exportin in the model; Supplementary Data 4). The disambiguated nuclear export module thus required no additional rate parameters beyond those specified in or corrected from Refs. [3, 4].

Model Addenda

Ref. [4] simulates a bipartite NLS from murine cap-binding protein p80 (NLS_{CBP80}), which binds to importin-α–importin-β with 10-fold higher affinity[16] than the NLS from SV40 T antigen ($NLS_{SV40Tag}$) used in the original model[3]. However, no information is provided about how NLS_{CBP80} was modeled. To retain the two-state binding kinetics of $NLS_{SV40Tag}$ (Eqn. 2), we assumed that $k_{on,1}$ and $k_{on,2}$ of NLS_{CBP80} were identical to $NLS_{SV40Tag}$ (Supplementary Data 4). We then reduced $k_{off,1}$ of NLS_{CBP80} to its lowest order of magnitude (0.01 s^{-1}) and fit $k_{off,2}$ to approximate the published affinity ($k_{off,2} = 2.5x10^{-4}\ s^{-1} \Rightarrow K_{D,eff} = 2.5$ nM ~ 2.4 ± 0.5 nM for NLS_{CBP80}[16]). Under these assumptions, import of NLS_{CBP80} cargo is predicted to occur at a faster initial rate and higher steady state compared to $NLS_{SV40Tag}$ cargo (Fig. SN2a,c). The predictions do not fully agree with Ref. [4], likely because of the million-fold correction in cargo affinity described earlier (see Model Corrigenda above). Nevertheless, the transport of NLS_{CBP80} cargo remains slower than the IBB domain of importin-α (NLS_{IBB}), a different type of cargo that binds directly to importin-β. Binding parameters for importin-β and NLS_{IBB} were reported in Ref. [3] (with the same $k_{on,2}$ errors as $NLS_{SV40Tag}$; Supplementary Data 4), but they were never used in that work. With the corrected rate parameters, we simulated import of NLS_{IBB} and observed faster initial import and a higher steady state compared to both $NLS_{SV40Tag}$ and NLS_{CBP80} (Fig. SN2). These predictions were qualitatively similar to Ref. [4].

Another omission of Ref. [4] was the reported abundance of "native" NES cargo akin to the native α–β cargo and native β cargo estimated in Ref. [3]. We circumvented this limitation by cross-referencing a database of confirmed NES-containing endogenous proteins[17] with a recent SWATH-MS dataset of absolute protein abundances in HeLa cells[18]. Aggregating the detectable NES-containing proteins yielded an estimate of 1 μM native NES cargo after rounding upwards. We adopted a similar approach to update the prior estimate of native β cargo by using two proteomic surveys of β-binding cargo[19, 20] along with the SWATH-MS resource in HeLa cells[18] to tabulate copy numbers per cell. Aggregating the detectable β-binding proteins yielded an estimate of 5 μM native β cargo after rounding upwards, a fivefold higher estimate than postulated in Ref. [3].

Last, we used the widely cited quantity of 3000 NPCs[21] per HeLa cell as the starting point for defining the initial concentration of nuclear pores in the perinuclear subcompartment. This number is far in excess the NPC counts where transport is predicted to become restricted and thus is not a sensitive initial condition in the model (Fig. SN2). Overall, despite considerable uncertainty in the NPC module of the earlier work[4], our implementation here retains the important characteristics of different NLS cargo types with conservative changes relative to the original modeling framework[3, 4].

Model Generalization

The earlier models[3, 4] were developed and tested in HeLa cells; thus, it was important to adapt the new model to B2:B1 cells. We stained trypsinized B2:B1 cells with wheat germ agglutinin and DAPI, segmented, and quantified cells as spheres to arrive at median cytoplasmic and nuclear volumes of $V_{cyt,B2:B1}$ = 1.45 pL and $V_{nuc,B2:B1}$ = 0.52 pL. Our own estimates for HeLa subcellular volumes ($V_{cyt,HeLa}$ = 2.55 pL; $V_{nuc,HeLa}$ = 0.87 pL) were larger than those and used in the Jarnac code of Ref. [3] but agree better with published values[22, 23]. To

adapt initial protein concentrations, we performed quantitative immunoblotting[24] on equal numbers of B2:B1 cells and HeLa cells (kindly provided by Dr. Ian Macara, Vanderbilt University) (Fig. SN3). Serial dilutions of lysate established a hyperbolic calibration curve relating arbitrary protein mass (*M*) to integrated band intensity. The differences in arbitrary protein mass were then scaled by the ratio of whole-cell volumes and the reported whole-cell HeLa concentration of the protein (C_{HeLa}) from Ref. [3] or Ref. [4]:

$$C_{B2:B1} = C_{HeLa} \frac{M_{B2:B1}/(V_{cyt,B2:B1}+V_{nuc,B2:B1})}{M_{HeLa}/(V_{cyt,HeLa}+V_{nuc,HeLa})} \quad (7)$$

Copy numbers per cell were generally comparable between B2:B1 cells and HeLa cells (Fig. SN3). However, the ~40% smaller volume of B2:B1 cells indicated that whole-cell concentrations of some species were as much as threefold higher (Table SN1). We extended this trend by increasing the estimated concentration of "generic transport receptors" for the HeLa cells[3] (3.6 μM) to 6 μM for B2:B1 cells (Supplementary Data 4).

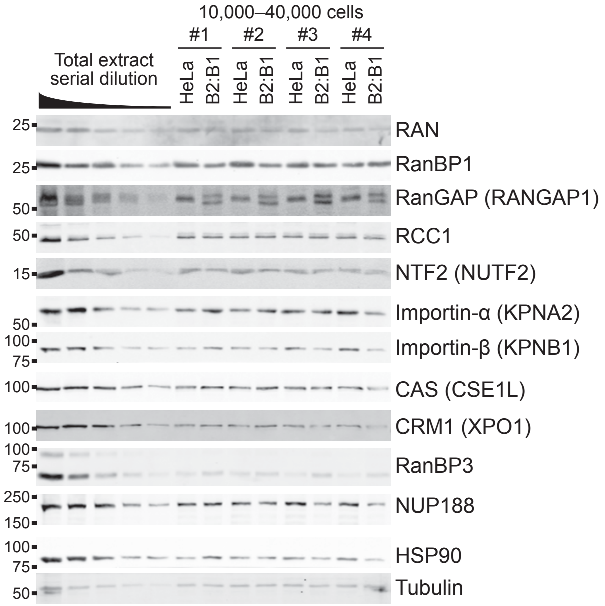

Fig. SN3. Quantitative immunoblotting of relative abundances between HeLa cells and B2:B1 cells. Common names from Riddick and Macara[1,2] are listed with the official gene name in parenthesis if different. NUP188 was used as a core nucleoporin to estimate the total number of NPCs, and HSP90 and Tubulin were used as loading controls.

Model Species	HeLa	B2:B1	Units
Ran	5 ± 0.8	5.2 ± 0.4	μM
RanBP1	2 ± 0.4	2.7 ± 0.6	μM
RanGAP	0.5 ± 0.03	0.9 ± 0.2	μM
RCC1	0.25 ± 0.01	0.3 ± 0.03	μM
NTF2	0.6 ± 0.07	0.8 ± 0.07	μM
Importin-α	1 ± 0.2	1.5 ± 0.2	μM
Importin-β	3 ± 0.9	2.8 ± 0.3	μM
Cas	3 ± 0.1	5.3 ± 0.9	μM
CRM1	0.3 ± 0.02	0.5 ± 0.08	μM
RanBP3	0.05 ± 0.01	0.15 ± 0.01	μM
Nuclear pore complexes	3000 ± 300	2600 ± 500	copies

Table SN1. Empirically adjusted protein concentrations for B2:B1 cells in the nucleocytoplasmic shuttling model according to per-cell relative copy number estimates (Fig. SN3) and differences in cell volume (Supplementary Data 4).

Supplementary References

1. Worsham MJ*, et al.* High-resolution mapping of molecular events associated with immortalization, transformation, and progression to breast cancer in the MCF10 model. *Breast Cancer Res Treat* **96**, 177-186 (2006).

2. Lopacinski AB*, et al.* Modeling the complete kinetics of coxsackievirus B3 reveals human determinants of host-cell feedback. *Cell Syst* **12**, 304-323 e313 (2021).

3. Riddick G, Macara IG. A systems analysis of importin-{alpha}-{beta} mediated nuclear protein import. *J Cell Biol* **168**, 1027-1038 (2005).

4. Riddick G, Macara IG. The adapter importin-alpha provides flexible control of nuclear import at the expense of efficiency. *Mol Syst Biol* **3**, 118 (2007).

5. Sauro HM*, et al.* Next generation simulation tools: the Systems Biology Workbench and BioSPICE integration. *OMICS* **7**, 355-372 (2003).

6. Hucka M, Finney A, Sauro HM, Bolouri H, Doyle J, Kitano H. The ERATO Systems Biology Workbench: enabling interaction and exchange between software tools for computational biology. *Pac Symp Biocomput*, 450-461 (2002).

7. Chelliah V*, et al.* BioModels: ten-year anniversary. *Nucleic Acids Res* **43**, D542-548 (2015).

8. Bischoff FR, Gorlich D. RanBP1 is crucial for the release of RanGTP from importin beta-related nuclear transport factors. *FEBS Lett* **419**, 249-254 (1997).

9. Catimel B*, et al.* Biophysical characterization of interactions involving importin-alpha during nuclear import. *J Biol Chem* **276**, 34189-34198 (2001).

10. von Appen A*, et al.* In situ structural analysis of the human nuclear pore complex. *Nature* **526**, 140-143 (2015).

11. Bui KH*, et al.* Integrated structural analysis of the human nuclear pore complex scaffold. *Cell* **155**, 1233-1243 (2013).

12. Ori A*, et al.* Cell type-specific nuclear pores: a case in point for context-dependent stoichiometry of molecular machines. *Mol Syst Biol* **9**, 648 (2013).

13. Ribbeck K, Gorlich D. Kinetic analysis of translocation through nuclear pore complexes. *EMBO J* **20**, 1320-1330 (2001).

14. Lindsay ME, Holaska JM, Welch K, Paschal BM, Macara IG. Ran-binding protein 3 is a cofactor for Crm1-mediated nuclear protein export. *J Cell Biol* **153**, 1391-1402 (2001).

15. Englmeier L, Fornerod M, Bischoff FR, Petosa C, Mattaj IW, Kutay U. RanBP3 influences interactions between CRM1 and its nuclear protein export substrates. *EMBO Rep* **2**, 926-932 (2001).

16. Marfori M, Lonhienne TG, Forwood JK, Kobe B. Structural basis of high-affinity nuclear localization signal interactions with importin-alpha. *Traffic* **13**, 532-548 (2012).

17. la Cour T, Gupta R, Rapacki K, Skriver K, Poulsen FM, Brunak S. NESbase version 1.0: a database of nuclear export signals. *Nucleic Acids Res* **31**, 393-396 (2003).

18. Liu Y*, et al.* Multi-omic measurements of heterogeneity in HeLa cells across laboratories. *Nat Biotechnol* **37**, 314-322 (2019).

19. Kimura M, Okumura N, Kose S, Takao T, Imamoto N. Identification of cargo proteins specific for importin-beta with importin-alpha applying a stable isotope labeling by amino acids in cell culture (SILAC)-based in vitro transport system. *J Biol Chem* **288**, 24540-24549 (2013).

20. Kimura M, Morinaka Y, Imai K, Kose S, Horton P, Imamoto N. Extensive cargo identification reveals distinct biological roles of the 12 importin pathways. *Elife* **6**, (2017).

21. Maul GG*, et al.* Time sequence of nuclear pore formation in phytohemagglutinin-stimulated lymphocytes and in HeLa cells during the cell cycle. *J Cell Biol* **55**, 433-447 (1972).

22. Puck TT, Marcus PI, Cieciura SJ. Clonal growth of mammalian cells in vitro; growth characteristics of colonies from single HeLa cells with and without a feeder layer. *J Exp Med* **103**, 273-283 (1956).

23. Zhao L, Kroenke CD, Song J, Piwnica-Worms D, Ackerman JJ, Neil JJ. Intracellular water-specific MR of microbead-adherent cells: the HeLa cell intracellular water exchange lifetime. *NMR Biomed* **21**, 159-164 (2008).

24. Janes KA. An analysis of critical factors for quantitative immunoblotting. *Sci Signal* **8**, rs2 (2015).
