# Supplementary material for: Nucleocytoplasmic transport of active HER2 causes fractional escape from the DCIS-like state
Source: Nat Commun. 2023 Apr 13;14:2110. doi: 10.1038/s41467-023-37914-x (PMC10102026; doi:10.1038/s41467-023-37914-x)
Supplement: Supplementary file 3 — Description of Additional Supplementary Files [file 41467_2023_37914_MOESM3_ESM.pdf]

Description of Additional Supplementary Files

File Name: Supplementary Data 1
Description: Predicted regulatory heterogeneities in 3D-cultured B2B1 cells minus AP heterodimerizer.

File Name: Supplementary Data 2
Description: Predicted regulatory heterogeneities in 3D-cultured B2B1 cells plus AP heterodimerizer.

File Name: Supplementary Data 3
Description: Transcript heterogeneities and estimated expression frequencies for high-priority targets and candidates for the DE phenotype.

File Name: Supplementary Data 4
Description: Initial conditions and rate constants for the systems model of nucleocytoplasmic transport.

File Name: Supplementary Data 5
Description: Proximity labeled proteins in B2B1 cells expressing BirA*-CSE1L or BirA*-NUP37.

File Name: Supplementary Data 6
Description: Genomic loci bound by chimeric EGFR in B2B1 cells with or without AP heterodimerizer.

File Name: Supplementary Data 7
Description: Cloning details, PCR primers, and antibodies.

File Name: Supplementary Movie 1
Description: Brightfield time lapse of B2B1 outgrowths treated with AP heterodimerizer for the indicated times. Scale bar is 100 μm.
