# Supplementary material for: Nucleocytoplasmic transport of active HER2 causes fractional escape from the DCIS-like state
Source: Nat Commun. 2023 Apr 13;14:2110. doi: 10.1038/s41467-023-37914-x (PMC10102026; doi:10.1038/s41467-023-37914-x)
Supplement: Supplementary file 12 — Reporting Summary [file 41467_2023_37914_MOESM12_ESM.pdf]

Corresponding author(s): Janes K.A.

Last updated by author(s): Mar 22, 2023

Reporting Summary

Nature Portfolio wishes to improve the reproducibility of the work that we publish. This form provides structure for consistency and transparency in reporting. For further information on Nature Portfolio policies, see our Editorial Policies and the Editorial Policy Checklist.

Statistics

For all statistical analyses, confirm that the following items are present in the figure legend, table legend, main text, or Methods section.

n/a	Confirmed	
☐	☒	The exact sample size (*n*) for each experimental group/condition, given as a discrete number and unit of measurement
☐	☒	A statement on whether measurements were taken from distinct samples or whether the same sample was measured repeatedly
☐	☒	The statistical test(s) used AND whether they are one- or two-sided<br>*Only common tests should be described solely by name; describe more complex techniques in the Methods section.*
☐	☒	A description of all covariates tested
☐	☒	A description of any assumptions or corrections, such as tests of normality and adjustment for multiple comparisons
☐	☒	A full description of the statistical parameters including central tendency (e.g. means) or other basic estimates (e.g. regression coefficient) AND variation (e.g. standard deviation) or associated estimates of uncertainty (e.g. confidence intervals)
☐	☒	For null hypothesis testing, the test statistic (e.g. *F*, *t*, *r*) with confidence intervals, effect sizes, degrees of freedom and *P* value noted<br>*Give P values as exact values whenever suitable.*
☒	☐	For Bayesian analysis, information on the choice of priors and Markov chain Monte Carlo settings
☒	☐	For hierarchical and complex designs, identification of the appropriate level for tests and full reporting of outcomes
☐	☒	Estimates of effect sizes (e.g. Cohen's *d*, Pearson's *r*), indicating how they were calculated

Our web collection on statistics for biologists contains articles on many of the points above.

Software and code

Policy information about availability of computer code

Data collection	Metamorph for Olympus (version 7.10.2.240), Odyssey (version 3.0), Aura Imaging Software (version 4.0.7), LAS X STELLARIS Control Software (version 4.4.0.24861), Bio-Rad CFX Manager 3.1 (version 3.1.1517.0823), EVOS DiamondScope (version 2.0.2094.0)
Data analysis	MATLAB (version R2022a), ImageJ (version 1.53a), FastQC (version 0.11.5), Bowtie2 (version 2.2.9), MACS2 (version 2.2.7.1), Bcl2fastq2 (version v2.20.0.422), https://github.com/JanesLab/NucCytoShuttle

For manuscripts utilizing custom algorithms or software that are central to the research but not yet described in published literature, software must be made available to editors and reviewers. We strongly encourage code deposition in a community repository (e.g. GitHub). See the Nature Portfolio guidelines for submitting code & software for further information.

Data

Policy information about availability of data

All manuscripts must include a data availability statement. This statement should provide the following information, where applicable:

- Accession codes, unique identifiers, or web links for publicly available datasets
- A description of any restrictions on data availability
- For clinical datasets or third party data, please ensure that the statement adheres to our policy

Raw and processed microarray data are available at the Gene Expression Omnibus (GSE214455; https://www.ncbi.nlm.nih.gov/geo/query/acc.cgi?acc=GSE214455 reviewer token: qrstiycanbalxgl). Raw and processed ChIP-seq data are available at the Gene Expression Omnibus (GSE216242; https://www.ncbi.nlm.nih.gov/geo/

query/acc.cgi?acc=GSE216242 reviewer token: uxmfcgayfbajjud).
Proximity labeling proteomics are available via ProteomeXchange (PXD038327; Username: reviewer_pxd038327@ebi.ac.uk Password: 7rdULh96).

Human research participants

Policy information about studies involving human research participants and Sex and Gender in Research.

Reporting on sex and gender	No human research participants were involved in this study.
Population characteristics	No human research participants were involved in this study.
Recruitment	No human research participants were involved in this study.
Ethics oversight	No human research participants were involved in this study.

Note that full information on the approval of the study protocol must also be provided in the manuscript.

Field-specific reporting

Please select the one below that is the best fit for your research. If you are not sure, read the appropriate sections before making your selection.

☒ Life sciences ☐ Behavioural & social sciences ☐ Ecological, evolutionary & environmental sciences

For a reference copy of the document with all sections, see nature.com/documents/nr-reporting-summary-flat.pdf

Life sciences study design

All studies must disclose on these points even when the disclosure is negative.

Sample size	No calculation was performed to pre-determine sample size, but n ≥ 4 provides 84% power to detect an effect size of 2.5 standard deviations from the mean.
Data exclusions	No data were excluded from any analysis.
Replication	For studies involving 3D culture (Fig. 1c, 2a-f, 3a-d, 5i, 7i, and 7m and Supplementary Fig. 1f, 2a-c, 2e-j, 2l, 3c), experiments were performed in biological quadruplicate and successfully replicated qualitatively on two separate days. For studies involving quantitative PCR (Fig. 2g-j and Supplementary Fig. 1c-e, 2k, 4a-g, 7b-e, 7g), experiments were performed in biological octuplicate and successfully replicated qualitatively on two separate days. For studies involving intraductal xenografts (Fig. 3e and Supplementary Fig. 3e,f), experiments were performed in biological sextuplicate on two separate days; no formal replication of the n = 12 study was performed, because the results were generally consistent between days and no batch effect was noticeable. For validation immunoblots (Fig. 2a, 2c-d, 2e-f, 5i and Supplementary Fig. 1a, 2a-c, 2e-i, 2l, 3b, 3d, 4i, 4k, 5a-b, 5d, 7f, 7i, 7l), experiments were performed in technical duplicate without replication because the experiments were confirmatory rather than exploratory. For quantitative immunoblots (Fig. 5d-g, 6f-g and Supplementary Fig. 1b), experiments were performed as single experiments and replicated on three separate days. For mass spectrometry studies (Fig. 5c), experiments were performed as single experiments without replication because the key findings were replicated by targeted quantitative immunoblots on three separate days (Fig. 5e, 5g). For quantitative immunofluorescence studies (Fig. 4b, 5h, 7g-h and Supplemental Fig. 4j, 4l, 5c, 7a, 7h, 7j-k), experiments were performed in biological duplicate and replicated on two separate days. For proximity ligation studies (Fig. 6h-j and Supplemental Fig. 6g-h) experiments were performed as single experiments and replicated on two separate days. For chromatin immunoprecipitation sequencing studies (Fig. 7b, 7d), experiments were performed as single experiemnts and replicated on two separate days. For chromatin immunoprecipitation qPCR studies (Fig. 7e), experiments were performed as single studies and replicated on five separate days.
Randomization	Mouse surgical order and left-right glands assignments for experimental-control injections were randomized at the start of each experiment. For all other experiments, randomization of pharmacologic interventions was performed by aliquoting the indicated genotypes into separate wells. Allocation of cell genotypes was not random, and covariates were controlled by comparing against a matched control genotype prepared concurrently with the experimental genotype.
Blinding	Investigators were not blinded to group allocation during data collection or analysis to avoid errors related to handling large numbers of linked-and-coded lists paired to the many experiments in this study.

Reporting for specific materials, systems and methods

We require information from authors about some types of materials, experimental systems and methods used in many studies. Here, indicate whether each material, system or method listed is relevant to your study. If you are not sure if a list item applies to your research, read the appropriate section before selecting a response.

Materials & experimental systems

n/a	Involved in the study
☐	☒ Antibodies
☐	☒ Eukaryotic cell lines
☒	☐ Palaeontology and archaeology
☐	☒ Animals and other organisms
☒	☐ Clinical data
☒	☐ Dual use research of concern

Methods

n/a	Involved in the study
☐	☒ ChIP-seq
☒	☐ Flow cytometry
☒	☐ MRI-based neuroimaging

Antibodies

Antibodies used

Supplementary Data 7

Validation

RRID:AB_309826 is validated for immunoblotting human Akt1>Akt2 by the manufacturer at a dilution of 1:1000.
RRID:AB_2315049 is validated for immunoblotting human Akt (P-Ser473) by the manufacturer at a dilution of 1:2000.
RRID:AB_2083165 is validated for immunoblotting human CLTC by the manufacturer at a dilution of 1:1000 and independently validated by CLTC knockdown in Fig. 5i.
RRID:AB_10865417 is validated for immunoblotting human CSE1L by the manufacturer at a dilution of 1:1000 and independently by CSE1L knockdown in Fig. 2e, and it is validated for immunocytochemistry by the manufacturer at a dilution of 1:1000.
RRID:AB_2246311 is validated for immunoblotting human EGFR by the manufacturer at a dilution of 1:1000 and independently validated by EGFR chimera overexpression in Supplementary Fig. 1a.
RRID:AB_330744 is validated for immunoblotting human ERK1/2 by the manufacturer at a dilution of 1:1000.
RRID:AB_2315112 is validated for immunoblotting human ERK1/2 (P-Thr202/Tyr204) by the manufacturer at a dilution of 1:2000.
RRID:AB_262044 is validated for immunoblotting ectopic FLAG by the manufacturer at a dilution of 1:1000 and independently validated at a dilution of 1:5000 by 3xFLAG overexpression in Fig. 2a, 2c-d and Supplementary Fig. 2a-c, 2e-i, 2l, 3b, 5a-b.
RRID:AB_300798 is validated for immunoblotting jellyfish GFP by the manufacturer at a dilution of 1:5000 and independently validated by GFP overexpression in Supplementary Fig. 4k, 7i), and it is validated for immunocytochemistry by the manufacturer at a dilution of 1:2000 and independently validated at a dilution of 1:500 by colocalization in Supplementary Fig. 4j.
RRID:AB_2191052 is validated for immunoblotting ectopic GluGlu by the manufacturer at a dilution of 1:1000, it is validated for immunoprecipitation by the manufacturer at a dilution of 1:50 and independently validated in GluGlu-expressing extracts in Fig. 6f, it is validated for immunocytochemistry at a dilution of 1:200 by colocalization in Fig. 7a, it is validated for proximity ligation assay at a dilution of 1:200 by inducible colocalization in Supplementary Fig. 6g,h, and it is validated for chromatin immunoprecipitation at 5 µg by IgG controls in Fig. 7b, 7d.
RRID:AB_398142 is validated for immunocytochemistry of human GM130 by the manufacturer at a range of application-specific dilutions and at a dilution of 1:200 in PMID: 29161592.
RRID:AB_307019 is validated for immunocytochemistry of ectopic HA by the manufacturer at a dilution of 1:100, and it is validated for proximity ligation assay at a dilution of 1:500 by inducible colocalization in Fig. 6j.
RRID:AB_390918 is validated for immunoblotting of ectopic HA by the manufacturer at a dilution of 1:2000 (50 ng/ml) and independently validated at a dilution of 1:2500 (40 ng/ml) by inducible co-immunoprecipitation in Fig. 6f, it is validated for immunocytochemistry by colocalization in Fig. 7a, and it is validated for proximity ligation assay at a dilution of 1:200 by inducible colocalization in Supplementary Fig. 6g,h
RRID:AB_2099242 is validated for immunoblotting overexpressed human HER2 (and predicted for rodent HER2) at a dilution of 1:1000 and independently validated by Erbb2 chimera overexpression in Supplementary Fig. 1a.
RRID:AB_2121235 is validated for immunoblotting human HSP90 at a dilution of 1:2000 in PMID: 25852189.
RRID:AB_397855 is validated for immunocytochemistry of human KPNA1 by the manufacturer at a range of application-specific dilutions and independently validated at a dilution of 1:200 by inducible downregulation in Fig. 7h.
RRID:AB_1860701 is validated for immunoblotting of human KPNA2 at an assay-dependent concentration and independently validated at a dilution of 1:1000 by inducible KPNA2 overexpression in Fig. 6f, it is validated for proximity ligation assay at a dilution of 1:500 by inducible colocalization in Fig. 6j.
RRID:AB_2787413 is validated for immunoblotting human KPNA2 by the manufacturer at a dilution of 1:1000.
RRID:AB_2133986 is validated for immunoblotting human KPNB1 by the manufacturer at a dilution of 1:5000 and independently validated at a dilution of 1:1000 by inducible KPNB1 overexpression in Fig. 6f, and it is validated for proximity ligation assay at a dilution of 1:500 by inducible colocalization in Fig. 6j.
RRID:AB_2737298 is validated for immunoblotting ectopic mCherry by the manufacturer at a dilution of 1:1000 and independently by mCherry overexpression in Supplementary Fig. 4k, 7i, and it is validated for immunocytochemistry at a dilution of 1:500 by colocalization in Supplementary Fig. 4j.
RRID:AB_61259 is validated for flow cytometry of human NGFR by the manufacturer and titrated at 1:400 dilution to yield the clones shown in Supplementary Fig. 7l.
RRID:AB_2157802 is validated for immunoblotting human NTF2 by the manufacturer at a dilution of 1:1000.
RRID:AB_1211263 is validated for immunoblotting human NUP160 by the manufacturer at a dilution of 1:2000.
RRID:AB_1850233 is validated for immunoblotting human NUP188 by the manufacturer at a dilution of 1:1000.
RRID:AB_10839500 is validated for immunoblotting human NUP37 at a dilution of 1:1000 by inducible overexpression in Supplementary Fig. 5b.
RRID:AB_628082 is validated for immunoblotting human p53 by the manufacturer at a dilution of 1:1000.
RRID:AB_2163120 is validated for immunoblotting human PDI by the manufacturer at a dilution of 1:1000.
RRID:AB_397730 is validated for immunoblotting human RAN by the manufacturer at a dilution of 1:5000.
RRID:AB_325804 is validated for immunoblotting human RanBP1 by the manufacturer at a dilution of 1:1000.
RRID:AB_2532281 is validated for immunoblotting human RanBP3 by the manufacturer at a dilution of 1:1000.
RRID:AB_2533099 is validated for immunoblotting human RanGAP by the manufacturer at a dilution of 1:1000.
RRID:AB_11178658 is validated for immunocytochemistry of human Rb (P-Ser807/811) by the manufacturer at a dilution of 1:1000.

RRID:AB_2039517 is validated for immunoblotting human RCC1 by the manufacturer at a dilution of 1:1000 (1 μg/ml).
RRID:AB_2238583 is validated for immunoblotting human S6 by the manufacturer at a dilution of 1:1000.
RRID:AB_10694233 is validated for immunoblotting human S6 (P-Ser240/244) by the manufacturer at a dilution of 1:1000.
RRID:AB_2620329 is validated for immunoblotting human Sec13 by the manufacturer at a dilution of 1:2000.
RRID:AB_2240087 is validated for immunoblotting human STAT1 by the manufacturer at a dilution of 1:1000.
RRID:AB_561284 is validated for immunoblotting human STAT1 (P-Tyr701) by the manufacturer at a dilution of 1:1000.
RRID:AB_390707 is validated for immunoblotting human TGFBR3 by the manufacturer at a dilution of 1:1000.
RRID:AB_10672056 is validated for immunoblotting human tubulin at a dilution of 1:20,000 in PMID: 25852189.
RRID:AB_2556564 is validated for immunoblotting ectopic V5 by the manufacturer at a dilution of 1:5000, and it is validated for immunocytochemistry by the manufacturer at a dilution of 1:500.
RRID:AB_309711 is validated for immunoblotting human vinculin at a dilution of 1:10,000 in PMID: 25852189.
RRID:AB_399312 is validated for immunoblotting human XPO1 at a dilution of 1:1000.
RRID:AB_2534088 is validated for immunocytochemistry of mouse IgG by the manufacturer at a dilution of 1:200.
RRID:AB_141780 is validated for immunocytochemistry of mouse IgG by the manufacturer at a dilution of 1:200.
RRID:AB_2535805 is validated for immunocytochemistry of mouse IgG by the manufacturer at a dilution of 1:200.
RRID:AB_2576217 is validated for immunocytochemistry of rabbit IgG by the manufacturer at a dilution of 1:200.
RRID:AB_2535850 is validated for immunocytochemistry of rabbit IgG by the manufacturer at a dilution of 1:200.
RRID:AB_2535813 is validated for immunocytochemistry of rabbit IgG by the manufacturer at a dilution of 1:200.
RRID:AB_621840 is validated for immunoblotting mouse IgG by the manufacturer at a dilution of 1:20,000.
RRID:AB_621842 is validated for immunoblotting mouse IgG by the manufacturer at a dilution of 1:20,000.
RRID:AB_621841 is validated for immunoblotting rabbit IgG by the manufacturer at a dilution of 1:20,000.
RRID:AB_621843 is validated for immunoblotting rabbit IgG by the manufacturer at a dilution of 1:20,000.
RRID:AB_10707008 is validated for immunoblotting chicken IgY by the manufacturer at a dilution of 1:20,000.
RRID:AB_1850023 is validated for immunoblotting chicken IgY by the manufacturer at a dilution of 1:20,000.

Eukaryotic cell lines

Policy information about cell lines and Sex and Gender in Research

Cell line source(s)	B2B1 cells were genetically engineered and subcloned from MCF10A-5E cells, which were originally isolated by the corresponding author (K.A. Janes) from MCF10A cells (female, ATCC). TM15c6 cells were provided by W.J. Muller and were originally derived from a female mammary tumor. HeLa cells (female, ATCC) used to calibrate the original nucleocytoplasmic transport model were provided by I.G. Macara.
Authentication	All cell lines were obtained from the original sources from which they were derived. No additional authentication was performed.
Mycoplasma contamination	Cells were confirmed negative for mycoplasma contamination.
Commonly misidentified lines (See ICLAC register)	No commonly misidentified cell lines were used in this study.

Animals and other research organisms

Policy information about studies involving animals; ARRIVE guidelines recommended for reporting animal research, and Sex and Gender in Research

Laboratory animals	SCID-beige (seven weeks old)
Wild animals	No wild animals were used.
Reporting on sex	Female, virgin SCID/beige mice (Envigo, 186). Only female mice were used because breast cancer is very rare in males.
Field-collected samples	No animal samples were collected in the field.
Ethics oversight	All animal work was done in compliance with ethical regulations under University of Virginia IACUC approval #3945.

Note that full information on the approval of the study protocol must also be provided in the manuscript.

ChIP-seq

Data deposition

☒ Confirm that both raw and final processed data have been deposited in a public database such as GEO.

☒ Confirm that you have deposited or provided access to graph files (e.g. BED files) for the called peaks.

Data access links *May remain private before publication.*	https://www.ncbi.nlm.nih.gov/geo/query/acc.cgi?acc=GSE216242 reviewer token: uxmfcgayfbajjud
Files in database submission	MinusAP_EGFRGluGlu_BiolRep1_R1.fastq.gz<br>MinusAP_EGFRGluGlu_BiolRep1_R2.fastq.gz<br>MinusAP_EGFRGluGlu_BiolRep2_R1.fastq.gz

MinusAP_EGFRGluGlu_BiolRep2_R2.fastq.gz
MinusAP_Input_BiolRep1_R1.fastq.gz
MinusAP_Input_BiolRep1_R2.fastq.gz
MinusAP_Input_BiolRep2_R1.fastq.gz
MinusAP_Input_BiolRep2_R2.fastq.gz
MinusAP_NaiveIgG_BiolRep1_R1.fastq.gz
MinusAP_NaiveIgG_BiolRep1_R2.fastq.gz
MinusAP_NaiveIgG_BiolRep2_R1.fastq.gz
MinusAP_NaiveIgG_BiolRep2_R2.fastq.gz
PlusAP_EGFRGluGlu_BiolRep1_R1.fastq.gz
PlusAP_EGFRGluGlu_BiolRep1_R2.fastq.gz
PlusAP_EGFRGluGlu_BiolRep2_R1.fastq.gz
PlusAP_EGFRGluGlu_BiolRep2_R2.fastq.gz
PlusAP_Input_BiolRep1_R1.fastq.gz
PlusAP_Input_BiolRep1_R2.fastq.gz
PlusAP_Input_BiolRep2_R1.fastq.gz
PlusAP_Input_BiolRep2_R2.fastq.gz
PlusAP_NaiveIgG_BiolRep1_R1.fastq.gz
PlusAP_NaiveIgG_BiolRep1_R2.fastq.gz
PlusAP_NaiveIgG_BiolRep2_R1.fastq.gz
PlusAP_NaiveIgG_BiolRep2_R2.fastq.gz
CON_GluGlu_rep1_narrow_summits.bed
CON_GluGlu_rep2_narrow_summits.bed
DIM_GluGlu_rep1_narrow_summits.bed
DIM_GluGlu_rep2_narrow_summits.bed

Genome browser session
(e.g. UCSC)

http://genome.ucsc.edu/s/paudelbb/hg38_miR205

Methodology

Replicates

Two biological replicates (independently prepared chromatin fractions) for cells treated with or without dimerizer for 24 hours.

Sequencing depth

15.7–31.8 million 75 bp paired-end reads

Antibodies

GluGlu Rabbit Cell Signaling 2448 RRID:AB_2191052 5 µg per ChIP

Peak calling parameters

Peaks were called using MACS2 (version 2.2.7.1) comparing immunoprecipitated chromatin with input chromatin using default settings.

Data quality

Read quality was assessed with FastQC (version 0.11.5), and reads were aligned to the human genome (GRCh38) using Bowtie2 (version 2.2.9) with default settings. Only uniquely mapped and non-duplicated reads were selected for further analysis.

Software

Peaks were called using MACS2 (version 2.2.7.1) comparing immunoprecipitated chromatin with input chromatin using default settings.
